# Local Mechanical Stimulation of Mardin-Darby Canine Kidney Cell Sheets on Temperature-Responsive Hydrogel

**DOI:** 10.3390/ijms13011095

**Published:** 2012-01-19

**Authors:** Ichiro Harada, Shunpei Yanagisawa, Katsuhiko Iwasaki, Chong-Su Cho, Toshihiro Akaike

**Affiliations:** 1 Department of Biomolecular Engineering, Graduate School of Bioscience and Biotechnology, Tokyo Institute of Technology, B-57, 4259 Nagatsuta-cho, Midori-ku, Yokohama 226-8501, Japan; E-Mails: syanagisawa@akaike-lab.bio.titech.ac.jp (S.Y.); iwasaki.k.ad@m.titech.ac.jp (K.I.); takaike@bio.titech.ac.jp (T.A.); 2 Research Institute for Agriculture and Life Sciences, Seoul National University, Seoul 151-921, Korea; E-Mail: chocs@plaza.snu.ac.kr

**Keywords:** mechanical stimulation, MDCK cell sheets, temperature-responsive hydrogel, collective cell migration, E-cadherin

## Abstract

Collective motion of cell sheets plays a role not only in development and repair, but also in devastating diseases such as cancer. However, unlike single-cell motility, collective motion of cell sheets involves complex cell-cell communication during migration; therefore, its mechanism is largely unknown. To elucidate propagation of signaling transduced by cell-cell interaction, we designed a hydrogel substrate that can cause local mechanical stretching of cell sheets. Poly (*N*-isopropyl acrylamide) (PNIPAAm) hydrogel is a temperature-responsive polymer gel whose volume changes isotropically in response to temperature changes below 37 °C. We designed a combined hydrogel substrate consisting of collagen-immobilized PNIPAAm as the local stimulation side and polyacrylamide (PAAm) as the non-stimulation side to assess propagation of mechanical transduction. Mardin-Darby canine kidney (MDCK) cells adhered to the collagen*-*immobilized PNIPAAm gel increased it area and were flattened as the gel swelled with temperature decrease. E-cadherin in these cells became undetectable in some domains, and actin stress fibers were more clearly observed at the cell base. In contrast, E-cadherin in cells adhered to the collagen*-*immobilized PAAm side was equally stained as that in cells adhered to the collagen*-*immobilized PAAm side even after temperature decrease. ERK1/2 MAPK activation of cells on the non-stimulated substrate occurred after partial stretching of the cell sheet suggesting the propagation of signaling. These results indicate that a change in the balance of mechanical tension induced by partial stretching of cell sheets leads to activation and propagation of the cell signaling.

## 1. Introduction

Collective motion of cell sheets plays a crucial role in many fundamental biological processes ranging from dynamical processes to those involved in homeostatic regulation such as morphogenesis, regeneration, and wound repair or devastating diseases such as cancer [1]. Many aspects of migratory behavior of cells can be conveniently observed *in vitro* by the wound healing scratch or comparable assay [2] in which the cells at the edge lead to coordinated movement of the cell sheet into free space. In epithelial cells, intercellular communication propagates according to the distance from the front row of cells to accomplish orchestrated movement of cells as a unit [3]. Because the collective motion of the cell sheet, unlike single-cell motility, involves complex cell-cell communication during migration, its mechanism is largely unknown.

Cell-cell adhesions remain tightly connected during collective migration, and moving cell sheets are thought to be maintained mainly by cadherin-based intercellular contacts. Adherens junctions, formed by homotypic interactions of cadherins between adjacent cells serve as attachment points for actin filaments, thereby allowing formation of a continuum of cytoskeleton between epithelial cells. Thus, the mechanical balance of cytoskeletal structure suggests existence of a key transducer of the mechanical signals [4]. Recent studies have demonstrated that ERK1/2 MAPK propagates from marginal cells to the interior of the cell sheet during collective motion [5,6]. Many reports suggest that motorgenic activity is propagated via cytoskeletal mechanical transduction; however, it is difficult to determine whether such motility signaling is actively induced by cell-cell transduction or is a consequence of a passive migratory signal from individual cells. Assessment of this question requires a method to analyze propagation of signaling among cells after imposing local mechanical stimulation on cell sheets without cellular migration.

In this study, we demonstrate stimulation of cultured cell sheets by local stretching using a combined hydrogel substrate that consisting of collagen*-*immobilized poly(*N*-isopropylacrylamide) (PNIPAAm) as the stimulation side and collagen*-*immobilized polyacrylamide (PAAm) as the non-stimulation side. PNIPAAm hydrogel is a temperature-responsive polymer gel whose volume changes isotropically in response to temperature changes below 37 °C [7,8]. It has several applications in biomedicine [9]. Previously, we demonstrated that PNIPAAm gel culture substrate could mechanically stretch cells by using its volume phase transition via temperature shift and that an increase in ERK phosphorylation was induced as a result of mechanical stimulation [10]. We expected that use of the NIPAAm/PAAm combined culture substrate would allow analysis of local mechanical stimuli, independently of individual migratory signaling.

Using this culture substrate, we found that the cell sheet on the NIPAAm side of the combined gel was mechanically stretched and that area of the adhered cell was increased and height was flattened. In addition, localization of E-cadherin between cell-cell adhesions in the cell sheet became inhomogeneous. Furthermore, induction of ERK phosphorylation in cells on PNIPAAm side was propagated to the cells on PAAm gel side, where mechanical stimulation was absent. Because the ERK activation in cells on PAAm side occurred within 15 min after the cells were stretched, it appeared that local mechanical stimulus was propagated by interaction via cell-cell communication and not by migration of individual cells.

## 2. Results and Discussion

### 2.1. Preparation of Collagen-Immobilized PNIPAAm/PAAm Combined Gel Culture Substrate

**Figure 1:**
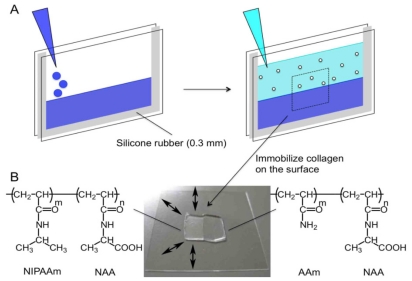
(**A**) Scheme for preparation of poly(*N*-isopropylacrylamide) (PNIPAAm)/polyacrylamide (PAAm) combined hydrogel. First, an aqueous solution of PAAm was poured between two glass plates, separated by a silicone rubber spacer. After polymerization of PAAm, an aqueous solution of PNIPAAm was poured onto PAAm and polymerized. The gels were then cut to a suitable size, and collagen was immobilized on the hydrogel surface; (**B**) Representative picture of the combined gel. To discriminate between the two gels, microbeads were added to either of the gels. The picture represents microbeads embedded within the collagen immobilized PNIPAAm gel (left). Only the left side of the PNIPAAm gel was able to swell and deform as indicated by the arrows.

In a previous study, we demonstrated that NIH 3T3 cells on fibronectin-immobilized temperature-responsive PNIPAAm gel were isotropically stretched when the gel swelled by changing the temperature [10]. To perform local stimulation of cell sheets, we designed a combined hydrogel (two-sided) substrate consisting of a PNIPAAm gel, which provided mechanical stimulation with temperature change, and a PAAm gel, which did not provide mechanical stimulation, as shown in Figure 1. We expected that propagation of local mechanical stimulation might be observed in cells on the PAAm gel because of the negligible temperature sensitivity of the PAAm gel. We found that PNIPAAm and PAAm gels were tightly bonded after the temperature was changed from 37 °C to 32 °C several times.

**Figure 2:**
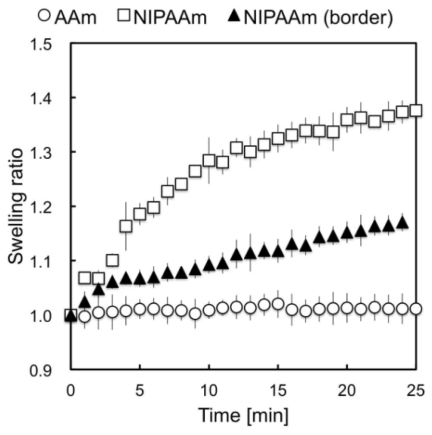
Swelling kinetics of combined gels. Temperature changes from 37 °C to 32 °C were applied to the gel (0 min). The swelling ratio against time was measured at five locations on the each section of the gel. Because the PNIPAAm did not swell evenly as a result of the effect of anchoring, whole-gel deformation was continuous. Open square; the collagen-immobilized PNIPAAm gel at a distanced from the border section. Closed triangle; the collagen-immobilized PNIPAAm gel side near the border section (within 100 μm). Open circle; the collagen-immobilized PAAm gel side at a distance from the border section. Values are mean ± SEM (*n* = 5).

Next, the swelling ratio of collagen*-*immobilized PNIPAAm was obtained by measuring the distance from the anchored section to the collagen*-*immobilized PAAm gel (Figure 2). For evaluating the swelling ratio, increased ratio of distance between randomly chosen micro-beads embedded within the gel was determined. Because the PNIPAAm gel and PAAm gel were tightly anchored, the swelling behavior of PNIPAAm was dependent on the location, especially near the border section. Thus, continuous whole-gel deformation was caused by swelling, whereas the remaining anchored section remained small swelling ratio. The swelling ratio of the collagen*-*immobilized PNIPAAm gel within 200 μm from the section anchored to the PAAm gel was less than 1.2 because of restriction of swelling to the parallel direction of the anchored surface. The swelling ratio of sections more than 200 μm from the border was 1.4, which was similar to that of the PNIPAAm gel alone under the same conditions. The collagen*-*immobilized PAAm gel near the anchored section was also slightly deformed by the effect of swelling of the collagen*-*immobilized PNIPAAm gel. Because deformation was small and mainly occurred in the *z*-axis direction, we were unable to estimate it quantitatively by measuring the distance between microbeads. However, as shown in Figure 2, the collagen*-*immobilized PAAm gel was not deformed at location more than 100 μm from the border section, where no deformation to *z*-axis was occured. Therefore, it can be said that cells on the collagen*-*immobilized PAAm gel side were not affected by direct deformation of the substrate if they were located more than 100 μm from the border section.

### 2.2. Observations of Change in Cell Shape on the collagen-Immobilized PNIPAAm Gel Side with Temperature Change

**Figure 3:**
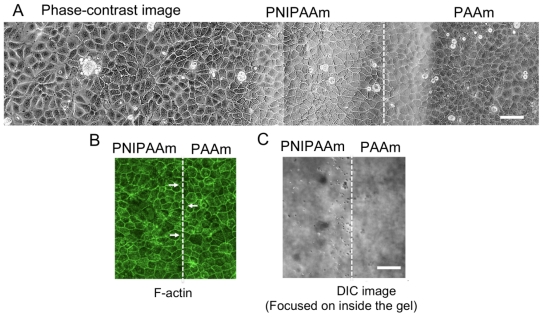
Representative pictures of Madin-Darby canine kidney (MDCK) cells on the border section of a combined gel after the temperature decreased. (**A**) Phase-contrast image of a large area, include PNIPAAm and PAAm gel side. Images were separately acquired at each location and converted to a single image; (**B**) MDCK cells stained with Alexa Fluor 488-conjugated phalloidin. No guidance effect between the two gels was observed, even in the cells in contact with both the gels (arrow); (**C**) Confirmation of the border of the two gels by focusing inside the gel by differential interference contrast (DIC). Microbeads were embedded only within the PNIPAAm side. White dashed line indicates the anchored section of the two gels. Scale bar: 50 μm.

To study the effect propagation of mechanical stimulus, we selected MDCK cells, which have been extensively used in studying the effect of cell-cell communication [2,11]. When these cells were cultured on the collagen*-*immobilized PNIPAAm/PAAm combined gel, they were kept adhered to the substrate, even after temperature was decreased (Figure 3A). The anchored section was easily confirmed by focusing inside of the gel because the beads were embedded within the PNIPAAm gel (Figure 3C). After temperature was decreased, area of the cells on the PNIPAAm side was larger than the cells on PNIPAAm gel side (Figure 3A). At the border section between the two gels, the cells were equally adhered compared with those far from the border section, and no guidance effect between the two different gels was observed by staining actin filaments in cells on the border section (Figure 3B).

**Figure 4:**
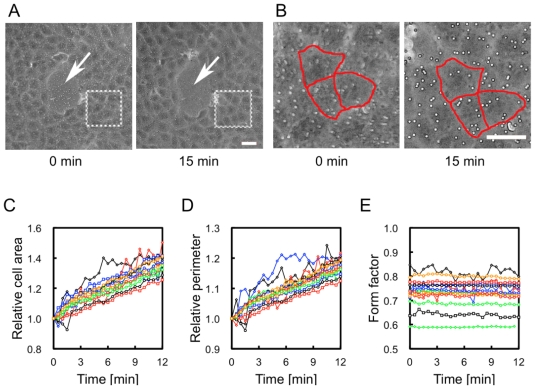
Observation and quantification of changes in cell shape during gel swelling. Cells cultured on the collagen-immobilized PNIPAAm gel side remained adhered during gel swelling. (**A**) Swelling of the gel was confirmed by enlargement of the hole in the cell sheet. The hole enlarged as the gel swelled (arrow), indicating that the entire area was equally swollen; (**B**) An example image of outline extraction for quantification of cellular shape. Images are magnified pictures of insert areas outlined in (**A**). Enlargement of each cell area was confirmed. Relative cell area (**C**), perimeter (**D**), and form factor (**E**) were plotted as a function of time after temperature decrease. Fifteen cells were randomly chosen and analyzed. Each color and shape on the plot corresponds to data of a single cell. Scale bar: 50 μm.

Next, we quantified morphological changes in cells on the NIPAAm side during temperature changes. MDCK cells on the PNIPAAm side remained adhered to the gel, and area of the cells was increase as the collagen*-*immobilized PNIPAAm gel swelled with temperature decrease (Figure 4A,B and supplementary Movie 1). To estimate the changes in cell shapes, we measured the area and perimeter of cells on the collagen*-*immobilized PNIPAAm side that were much more than 200 μm from the border section. As shown in Figure 4C,D, both the area and perimeter of cells increased as the gel swelled. To check whether morphological changes were isotropic, we calculated the form factor (Figure 4E). The form factors did not change during gel swelled, indicating that the cells retained their circularity during stretching deformation. Because an increase of area and perimeter were consisted to the swelling ratio of the gel, e.g., distances of micro-beads embedded in the gel, an observation of the cellular shape on PNIPAAm side (Figure 3A) was consequence of stretching cells according to the gel swelling after temperature decrease.

### 2.3. Observation of Adherens Junctions

**Figure 5:**
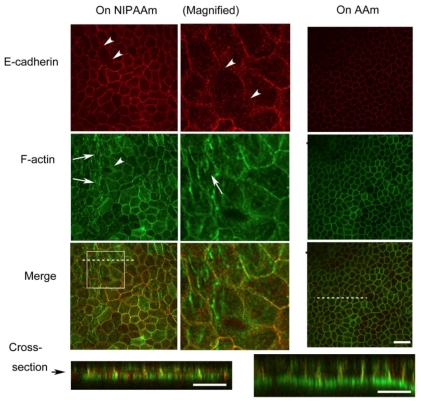
Representative picture of immunostained cells on each gel section. The center column shows magnified images of insert areas outlined in the merged image of PNIPAAm. Images were obtained by confocal microscopy. Location of cross-section is indicated by a white dashed line in the merged picture. Cells on the collagen-immobilized PNIPAAm gel side were clearly larger in cellular area and flattened. In some domains, obvious F-actin (arrow) and/or vanished E-cadherin (arrow head) were observed. Note that all F-actin or E-cadherin images were observed in the same condition, e.g., pinhole size, scanning rate and gain of photon counter. E-cadherin in the cells on the collangenimmobilized PNIPAAm was more brightly stained compared with that in cells on the collagen-immobilized PAAm side. Scale bar: 50 μm.

Because of the gel thickness (more than 0.3 mm) and was not tightly anchored to the bottom of a dish, we were unable to observe fluorescent live cell images under high magnification with our inverted microscope. We therefore stained actin-filaments and performed immunostaining of E-cadherin in fixed MDCK cell sheets after temperature decrease and observed for structural changes in the cytoskeleton and adherens junction. Figure 5 shows results of the immunostaining of cells on PNIPAAm and PAAm section after 15 min of temperature decrease from 37 °C to 32 °C. Interestingly, although the cellular periphery was clearly observed by phase-contrast microscopy (Figure 3A), the E-cadherin of the cells adhered to the collagen*-*immobilized PNIPAAm gel as well as actin between the cells in some domains was undetectable (Figure 5, arrowhead). E-cadherin in the cells adhered to the collagen*-*immobilized PAAm side was equally stained, indicating that adherens junctions in the cells were maintained 15 min after temperature decrease from 37 °C to 32 °C. Basal actin stress fibers were more clearly observed in the cells on the PNIPAAm gel side as we previously observed by NIH 3T3 cells. In addition, the height of the cells on PNIPAAm side decreased after the PNIPAAm substrate swelled and polarity in the *z*-direction was disappeared whereas it was retained in the cells on the PAAm side (Figure 5, cross-section). These observations suggested that inhomogeneity of cell-cell interaction mediated by E-cadherin in cells on the PNIPAAm side was caused by stretching stimulation, although such inhomogeneity of cytoskeletal structure was not propagated to the non-stimulated section.

### 2.4. Observation of ERK1/2 Signaling and Initial Propagation

**Figure 6:**
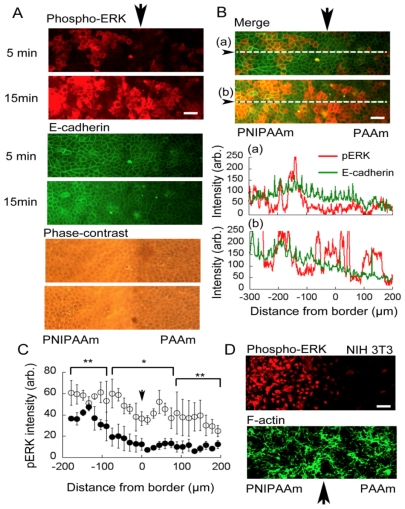
(**A**) Immunostaining of phosphorylated ERK1/2 and E-cadherin. Activation of ERK1/2 occurred within 5 min of the temperature change in the cells cultured on the collagen-immobilized PNIPAAm side. After 15 min, activation of ERK1/2 had propagated to the cells on the collagen-immobilized PAAm side include those more than 100 μm from the border section. E-cadherin of cells on collagen-immoblized PNIPAAm side was stained more strongly than that in cells on the collagen-immobilized PAAm side, although the staining pattern was not propagated. The black arrows indicate the border between the two gels. Scale bar: 50 μm; (**B**) Merged image of phospho-ERK and E-cadherin on (**A**). (a) and (b) are one pixel width intensity profiles on dashed line in each 5 min and 15 min image. The X-axis denotes the location of the combined gel, zero represents the border section, minus sign represent the PNIPAAm side, and plus sign represent PAAm gel side. No particular correlation intensity between phospho-ERK and E-cadherin was observed; (**C**) Fluorescence intensity profiles of phosphorylated ERK1/2. Closed circle; after 5min after temperature decrease. Open circle, after 15min of temperature decrease. Profiles are averages of three different section images from each of three independent experiments. Vales are mean ± SEM (*n* = 3). Statistical analysis between 5 min and 15 min images of each correspond plots were * *p* < 0.01, ** *p* < 0.05; (**D**) Immunostaining of phosphorylated ERK1/2 of NIH 3T3. Even after 15 min of a temperature decrease, ERK1/2 phosphorylated cells were few on PAAm side.

It has been previously demonstrated that ERK1/2 signaling was propagated because of collective motion of cell sheets after scratching assay [5]. Because migration of the cells as a result of mechanical stimuli was not observed within 15 min of temperature decrease, we investigated whether propagation of ERK activity propagation could be induced by local mechanical stimulation without cellular migration. We performed immunostaining for phosphorylated ERK1/2 after stretching cell sheets on the combined gel. Figure 6A shows an example of the pattern of ERK1/2 activation. We found that ERK1/2 phosphorylation in cells on the collagen*-*immobilized PNIPAAm side had increased after 5 min as reported previously [10] and that ERK1/2 phosphorylation had propagated to the cells on the collagen*-*immobilized PAAm side after 15 min (Figure 6A). As shown in Figure 6B, intensity signal profiles of phosphorylated ERK1/2 and E-cadherin were different, indicate staining pattern of phosphorylated ERK1/2 was independent from signal of stained E-cadherin. We estimated expansion of the activating profile by measuring fluorescence intensity against distance from border section according to an explanation in Experimental Section (Figure 6C). Fifteen minutes after temperature decrease, phosphorylated ERK1/2 was propagated to the cells on the PAAm side even more than 100 μm from the anchored section. When the same experiment was performed with NIH 3T3 fibroblast, we could not detect propagation of ERK1/2 phosphorylation in the cells on PAAm side after 15 min of temperature decrease (Figure 6D). The result indicate adherens junction involves E-cadherin interaction is necessary for propagation of activating ERK1/2.

**Figure 7:**
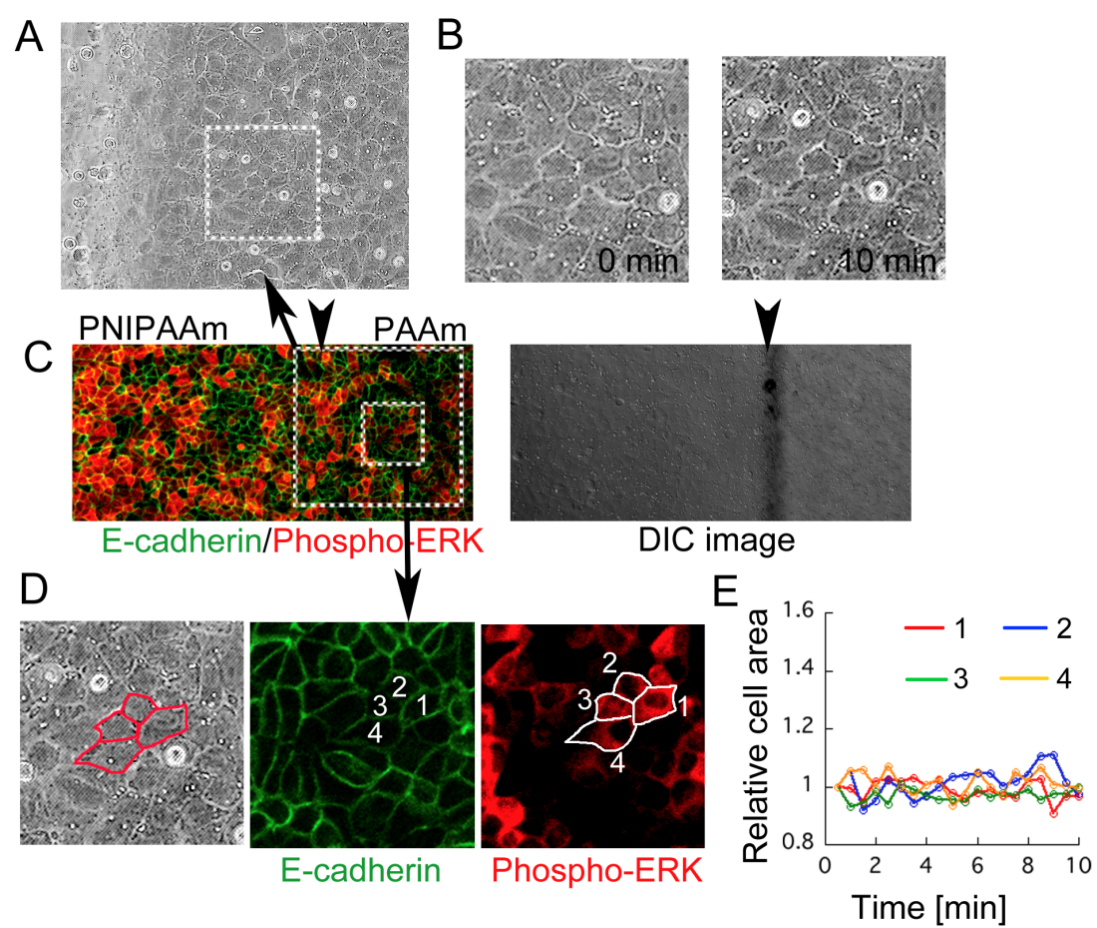
(**A**) Representative picture of the cells on near the border section after 10 min temperature decrease, which used for video-microscopy (supplementary Movie 2). The anchored section is left edge of the image; (**B**) Representative images show in magnified picture of 0 min and 10 min of temperature decrease for insert areas outlined in (**A**). No particular deformation of cells was observed; (**C**) Immunostaining of phosphorylated ERK1/2 and E-cadherin of cells on an around border section used in the video-microscopy. Arrowheads indicate the anchored section of two gels, confirmed by differential interference contrast (DIC) image. Large insert area indicates the section observed by video microscopy (**A**) and smaller insert area is enlarged to (**D**); (**D**) An example images of cellular shape for quantification from video-microscopy. Cellular area was estimated from sequential images acquired by video-microscopy after confirming cells were phosphorylate ERK positive; (**E**) Relative cell areas were plotted as a function of time after temperature decrease. Four phosphrylate ERK positive cells were chosen and analyzed. No particular deformation was observed as compared to cells on NIPAAm side (Figure 4C). Each color and shape on the plot corresponds to data of a single cell numbered of (**D**). Scale bar: 50 μm.

To assess whether ERK1/2 activated cells on the PAAm side were deformed or not, we performed video-microscopy and immunostaining of same cells on PAAm side near the border section (Figure 7 and supplementary Movie 2). We estimated a deformation of the cells by the cellular area. Although some of the cells on PAAm side were positively stained by anti-phospho ERK1/2 (Figure 7C), their deformation was undetectable by our estimation as compared to the deformation of the cells on PNIPAAm side (for example Figure 4B). Thus, it can be suggested that ERK1/2 activation in the cells on PAAm side was not induced by direct large deformation of the cells, but more microstructure change of the cells can be speculated. ERK1/2 is known to be activated by direct mechanical stimulation from adherent extracellular matrix signaling mediated by integrins [12]. Propagation of ERK1/2 signaling in the cells may therefore be attributed to the mechanical stimulation. In summary, these results indicate that a change in the balance of mechanical tension mediated by cell-cell adhesion induced by local stretching of cell sheets leads to activation and propagation of the ERK signaling.

Interestingly, as shown in Figure 6A,B, E-cadherin in cells on the collagen*-*immobilized PNIPAAm side was always more brightly stained than that in cells on the collagen*-*immobilized PAAm side, although the staining intensity was inhomogeneous. The mechanism of this phenomenon is not clear at present. However, several possibilities can be suggested. One possibility is that accumulation of E-cadherin in the cells was directly increased by stretch stimulation to the adherens junction. It was recently reported that activation of α-catenin, the scaffold protein of the intracellular domain of E-cadherin, acted as a mechanical sensor because the protein was partially unfolded by mechanical stimuli [13], suggesting that the increase in accumulated E-cadherin is because of such mechanical activation. Another possibility is a change in the conformation of adherens junctions because an anti-body was used as the antigen of the extracellular domain of E-cadherin and accessibility was changed as a result of change in adherens structure. However, clarification of this mechanism will require studies using a fluorescent live cell imaging technique.

## 3. Experimental Section

### 3.1. Cells and Reagents

MDCK cells were cultured in a tissue culture dish containing Dulbecco’s modified Eagle medium (DMEM, Nacalai Tesque) supplemented with 10% FBS and antibiotics (50 U/mL penicillin and 50 mg/mL streptomycin). The cell culture dish was maintained in a humidified atmosphere of 95% air and 5% CO_2_ at 37 °C. Collagen Type I was purchased from KOKEN (Tokyo, Japan). Anti-E-cadherin (DECMA-1) was obtained from Sigma, and anti-phospho-ERK (Thr202/Tyr204) (E-10) was provided by Cell Signaling Technology. Alexa Fluor 488 conjugated phalloidin was purchased from Invitrogen.

### 3.2. Preparation of Collagen-Immobilized PNIPAAm/PAAm Combined Hydrogel

*N*-acryloyl alanine (NAA) was prepared as previously described in [10]. PNIPAAm/PAAm combined hydrogel was prepared as follows: A solution of acrylamide (AAm) and NAA dissolved in distilled water with a mixture of AAm, NAA, *N,N′*-methylenebisacrylamide (BIS), 2-amino-2-hydroxymethyl-1,3-propanediol (Tris), *N,N,N′,N″*-tetramethylethyenediamine (TEMED), and 10% ammonium persulfate (APS) (950 mM: 50 mM: 10 mM: 50 mM: 10 μL/mL: 5 μL/mL) were poured between two glass plates (76 mm × 52 mm, 1.5-mm thickness) separated by a silicone spacers (0.3-mm thickness) and polymerized for 1 h. NIPAAm dissolved in distilled water with the mixture of NIPAAm, NAA, BIS, Tris, TEMED and 10% of APS (970 mM: 30 mM: 10 mM: 30 mM: 1 μL/mL: 5 μL/mL) was poured between the plates and polymerized for 1 h. Polystyrene (PS) latex beads (2 μm diameter; Polyscience) were also added to the above solution to visualize swelling behavior and the borderline between the two gels using optical microscopy. After swelling in phosphate-buffered saline (PBS) for 1 h, pieces of gel (1 × 2 cm) were cut from the slab and washed with PBS to remove the unreacted monomers. Collagen was immobilized on the gel surface according to a previously described method [10] with some modification. In brief, carboxyl groups of the gels were activated for 30 min at 25 °C with 1-ethyl-3(3-dimethylaminopropyl)-carbodiimide (EDAC) and *N*-hydroxysuccinimide (NHS) in MES buffer (0.1 M MES; 0.5 M NaCl, pH 6.1). The gels were then reacted with 0.1 mg/mL of collagen in HEPES buffer (0.5 M HEPES, pH 9.0) for 12 h at 4 °C, followed by washing in PBS at 4 °C. The gels were sterilized by exposure to UV for 30 min for sterilization. Before plating the cells, the gels were equilibrated in DMEM for 12 h at 4 °C.

### 3.3 Measurement of Temperature-Sensitivity of the Collagen-Immobilized PNIPAAm/PAAm Combined Hydrogel

The collagen-immobilized PNIPAAm/PAAm combined hydrogel was kept at 37 °C for 12 h. The gels were placed in a 35-mm dish containing 2.5 mL of cell culture medium. After the gel was transferred from 37 °C to 32 °C in a chamber (Cytomotion, Altair Corporation), time-lapse images of beads embedded within the gels were recorded with a charge-coupled device (CCD) camera (Penguin 600CL, Pixera) equipped with an optical microscope (IX70, OLYMPUS). The PS latex beads located beneath the gel surface (within 50 μm from surface) were clearly observed in the same focal plane during gel swelling. Distances between pairs of randomly chosen two beads were measured using ImageJ (http://rbs.info.nib.gov/imageJ) [14]. Swelling ratios were measured at five different locations of each PNIPAAm gel, PAAm gel, and PNIPAAm gel near the border section between the two gels.

### 3.4. Observation and Quantification of Changes in Cell Shape

MDCK cells were plated at a density of 5.0 × 10^5^ cells/cm^2^ on a collagen-immobilized PNIPAAm/PAAm combined hydrogel and cultured for 24 h to the confluence in cell density. After the gel was transferred from 37 °C to 32 °C in a chamber (Cytomotion) equipped on a microscope stage (IX70), changes in cell shape accompanying gel swelling were recorded using a CCD camera (Penguin 600CL). Each cell contour was traced with a pen tablet (WACOM), and the projected area and perimeter of 15 cells were quantified using ImageJ. The form factor, an index of the circularity of cells, was calculated as 4π × (area)/(perimeter)^2^.

### 3.5. Immunostaining and Quantification

After change in temperature change for 5 min or 15 min, each sample was fixed with PBS containing 4% (v/v) formaldehyde for 20 min and permeabilized with PBS containing 0.5% (v/v) Triton X-100 for 3 min. The cells were incubated for 30 min in blocking one (blocking buffer: Nacalai Tesque). The samples were exposed to an anti-E-cadherin antibody and anti-phospho-ERK antibodies for 1 h, followed by incubation with an Alexa Fluor 448-conjugated secondary antibody for anti E-cadherin, Alexa Fluor 555-conjugated secondary antibody for anti-phospho ERK and Alexa Fluor 488-conjugated phalloidin (Invitrogen) for 1 h. The samples were washed thrice with Tris-buffered saline (137 mM of NaCl, 2.68 mM of KCl, and 25 mM of Tris, pH 7.4). Images were obtained using a confocal microscope (A1; Nikon) equipped with CFI Plan DLL 10× and 40× objective lenses.

For fluorescence intensity analysis, all the images were acquired as 8-bit grayscale images under same conditions of sensitivity and exposure time. The intensity profile, which is perpendicular to the anchored section of the two gels, was calculated from the images using ImageJ software. The obtained images were sliced into columns of 25-pixel width parallel to the anchored section (175-pixel for 100 μm). The average intensity of each sliced 8-bit gray scale images was plotted against the distance from the anchored section. The results of three sections of each sample from all the three individual experiments were then averaged after confirming that their anchored sections were equally coordinated.

### 3.6. Statistical Analysis

Statistical significance was measured by a two-tailed Student’s *t*-test.

## 4. Conclusion

In this study, we designed a new combined hydrogel substrate consisting of collagen*-*immobilized PNIPAAm as the local stimulation side and collagen*-*immobilized PAAm as the non-stimulation side, which could locally stimulate the cells in the cell sheet by stretch force. Using this new-programmable material, we found that ERK1/2 activation was propagated by local mechanical stimuli.

## Supplementary Material

Supplementary Movie 1 Morphological change of MDCK cells during temperature decrease. Images were acquired at one minute interval and the movies was converted to 7 flames per seconds. Enlargement of the hole in the cell sheet as well as cells area can be confirmed.

Supplementary Movie 2 Morphological change of MDCK cells on the PAAm gel side near the anchored section of two gel during temperature decrease. Images were acquired at thirty seconds interval and the movies was converted to 7 flames per seconds. In the beginning of the movie, cells, which of analysis results shown Section 2.4 were denoted as traced outline. At end of movie, immunostaining of phosphorylated ERK1/2 (red) and E-cadherin (green) of same cells are represented.
